# Late successional tree species in Guam create biogeochemical niches

**DOI:** 10.1080/19420889.2019.1619437

**Published:** 2019-05-25

**Authors:** Thomas E. Marler

**Affiliations:** College of Natural and Applied Sciences, University of Guam, Mangilao, Guam, USA

**Keywords:** Artocarpus mariannensis, biogeochemical heterogeneity, Elaeocarpus joga, Serianthes nelsonii

## Abstract

The soils beneath and surrounding mature *Artocarpus mariannensis, Elaeocarpus joga*, and *Serianthes nelsonii* trees were studied in northern Guam limestone forests to determine the role of these trees in maintaining spatial heterogeneity of biogeochemistry. The soils beneath *S. nelsonii* were nutrient-enriched compared to soils away from *S. nelsonii*. The soils beneath *A. mariannensis* were depauperate for some nutrients or were not different from the soils away from *A. mariannensis* for other nutrients. The soils beneath *E. joga* exhibited increases in some nutrients such as nitrogen, carbon, and phosphorus, but decreases in other nutrients such as potassium and calcium when compared to the soils away from *E. joga* trees. These three tree species influenced spatial heterogeneity in soil nutrient status in the order *A. mariannensis* < *E. joga* < *S. nelsonii* and their presence added greatly to surface soil heterogeneity. Iron, manganese, and pH exhibited the least variation within the paired sites. Calcium, magnesium, potassium, and zinc exhibited the greatest variation among the paired sites. These findings indicate that continuing loss of these trees from Guam’s forests will diminish the associated biogeochemical spatial heterogeneity.

## 1. Introduction

Late successional tree species in the forests of the Mariana Island Archipelago have been in a state of decline for many years. Three of these species are *Artocarpus mariannensis* Trécul, *Elaeocarpus joga* Merr., and *Serianthes nelsonii* Merr. The legume *S. nelsonii* that is endemic to the two southernmost islands of the archipelago, was already noted as rare when it was first described, and is currently listed as critically endangered. [1] The threats to these species are similar to those of the other declining tree species, and efforts are underway to better understand these threats. A failure to regenerate and attrition of the remaining trees due to ongoing anthropogenic activities are some of the relevant conservation concerns. The contemporary forests are characterized by a skewed population age structure toward large, mature individuals. [2–5]

By the time one of the trees of these species occupies the mature emergent forest canopy, it has been influencing the habitat niche for decades, and is therefore ideal for studying the spatial role in ecosystem services. Long-lived tree species may increase ecosystem spatial chemical heterogeneity by orchestrating many biotic and abiotic factors. [6–9] Some of these include chronic extraction and sequestration of soil elements in plant organs, the extent of element resorption prior to organ senescence, litter quality effects on organic matter lability, and local amplification or suppression of soil microorganisms. The direct study of tree-specific soil legacy effects is a subset of literature on the Janzen–Connell model [10,11] and the literature on plant-soil feedbacks. [12] In addition to residual effects on essential nutrients, these fields of study include allelochemicals and pathogens, and collectively may change plant community dynamics and nutrient cycling. While many of the case studies have focused on soils within large mono-specific stands, the influence of individual large canopy trees on biogeochemistry can be substantial, even in biodiverse tree communities. [13,14]

The niche properties of substrates associated with the focal tree species in comparison with nearby substrates away from the tree were determined by collecting soil samples associated with mature trees. My objective was to use established niche chemistry protocols to determine the soil chemical traits beneath the canopy of these three species and to compare these traits to the soils nearby but outside the influence of the focal tree species. Habitats dominated by legume species are known to exhibit soil nutrient relations that differ from non-legume habitats, [15–17] so I predicted the localized differences in soil chemical traits beneath the *S. nelsonii* tree would be greater in contrast with the general community traits than the other two species.

## 2. Materials and methods

The 17 study sites were located along the perimeter of the northern calcareous plateau of Guam within a longitudinal range of E 144.814° to 144.929° and a latitudinal range of N 13.464° to 13.649°. The tropical wet climate is Af under the Köppen-Geiger classification. The karst soils formed in slope alluvium, loess, and residuum overlying limestone (Clayey-skeletal, gibbsite, nonacid, isohyperthermic Lithic Ustorthents). [18] The calcareous plateau has a maximum height of 180–185 m above sea level. The forest type has been classified as “typhoon forests” due to tropical cyclone frequency. [19] The close proximity of paired sampling sites ensured homogeneous geological, edaphic, and disturbance histories for each of the 17 paired sites.

Soil samples were collected from the *S. nelsonii* site in Aug 2015 and from the sites for the other two species in Aug 2017. Soil-sampling methods were patterned after Marler and Krishnapillai. [20] The eight *A. mariannensis* and eight *E. joga* trees were mature components of the emergent canopy. Four transects were oriented in cardinal directions for each tree. Two sampling locations along each transect underneath each tree canopy included the location 1 m inside the periphery of the canopy, and half the distance between the bole and the periphery of the canopy. These eight subsamples were combined into one sample for each *A. mariannensis* and *E. joga* tree and defined as “home” soils. There were eight trees for these two species. Only one known mature *S. nelsonii* tree remains on Guam, so the eight home subsamples were not combined for these species and were treated as individual replications. Samples were also collected at fixed distances of 30 m and 60 m on each transect for a total of eight subsamples which were defined as “away” soils. If the prescribed site was within close proximity to a second tree of the same species, the location was moved closer or farther to ensure all away samples were not influenced by the focal species. Treatment of the replications was the same as for home soils. The litter layer was carefully brushed away to expose the mineral layer. One depth increment of 0–10 cm were sampled.

The soil for each replication was separated into three batches. One batch was dried at 50°C then total carbon (C) and nitrogen (N) contents were determined by dry combustion (FLASH EA1112 CHN analyzer; Thermo Fisher, Waltham, Mass., USA). Extractable essential nutrients other than phosphorus (P) were quantified following digestion with diethylenetriaminepentaacetic acid. [21] Analysis was by inductively coupled plasma optical emission spectrometry (Spectro Genesis; SPECTRO Analytical Instruments, Kleve, Germany). Available P was determined by the Olsen method. [22] Soil reaction was determined from saturated paste with an Accumet AB200 meter (Thermo Fisher Scientific, Singapore). A second batch of the fresh soil was analyzed for nitrate and ammonium following 2M potassium chloride extraction. [23] The third batch of fresh soil was incubated in situ for 25 days using the buried bag method. [24] Nitrate and ammonium were quantified at the end of the incubation period.

Several derived variables were calculated. Net nitrification rate was calculated by subtracting initial from final nitrate concentration and dividing by the incubation period. Net mineralization was calculated by subtracting initial nitrate plus ammonium from final nitrate plus ammonium concentration and dividing by the incubation period. Percent available nitrogen was calculated as ((nitrate+ammonium)/total nitrogen)*100. The stoichiometric relationship between carbon and nitrogen was quantified as C/N.

Most of the response variables did not meet parametric requirements primarily because of similarity among home soils and heterogeneity among the away soils. Therefore, the Mann-Whitney *U* test [25] was employed to determine the differences between home and away soils, as this test does not require any assumption about distribution of the data.

## 3. Results

### 3.1. Artocarpus mariannensis

**Figure 1.**
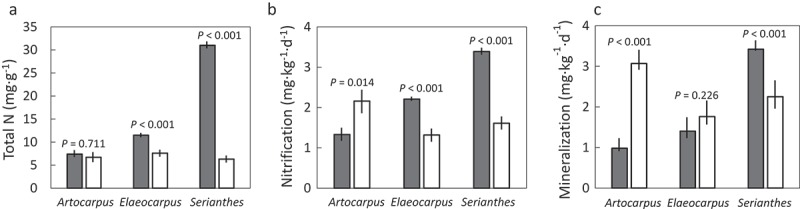
The influence of proximity to *Artocarpus mariannensis, Elaeocarpus joga*, or *Serianthes nelsonii* trees on soil chemical traits in karst soils on Guam. Home soils (shaded bars) were collected underneath the trees, and away soils (white bars) were collected 30 m and 60 m from focal trees. (a) Total nitrogen; (b) Net nitrification; and (c) Net mineralization. *P*-value determined by Mann-Whitney *U* test.

The soils away from mature *A. mariannensis* trees contained greater concentrations of P, potassium (K), calcium (Ca), magnesium (Mg), and manganese (Mn) than the soils beneath the trees (Table 1), according to the Mann-Whitney *U* test. The soils beneath *A. mariannensis* trees contained greater concentrations of copper and zinc than soils away from these trees. Ammonium, C, N, iron (Fe), nitrate, and pH were not influenced by sampling location (Table 1, Figure 1). Copper (Cu) and zinc (Zn) were the only elements that were increased by the presence of an *A. mariannensis* tree. The derived variables percent available N and C/N were also not influenced by sampling location (Table 1). The behavioral variables net nitrification and net mineralization were not influenced by proximity to *A. mariannensis* trees (Figure 1).

**Table 1.** Proximity to *Artocarpus mariannensis* trees and soil chemical traits in Guam. Home soils were collected underneath mature trees. Away soils were collected 30 m and 60 m from focal trees. Means ± SE, n = 8.

Soil trait	Home	Away	Significance
pH	7.7 ± 0.1	7.5 ± 0.1	0.226^1^
Total carbon (mg·g^−1^)	107.0 ± 5.2	97.3 ± 16.0	0.101
Phosphorus (µg·g^−1^)	39.2 ± 3.7	103.5 ± 18.1	<0.001
Potassium (µg·g^−1^)	34.1 ± 4.0	142.8 ± 14.7	<0.001
Calcium (mg·g^−1^)	8.7 ± 0.4	15.6 ± 2.3	<0.001
Magnesium (µg·g^−1^)	484.3 ± 29.6	828.8 ± 61.4	<0.001
Manganese (µg·g^−1^)	13.1 ± 0.8	118.3 ± 17.6	<0.001
Iron (µg·g^−1^)	13.0 ± 1.1	21.6 ± 5.9	0.066
Copper (µg·g^−1^)	2.3 ± 0.2	1.6 ± 0.1	0.004
Zinc (µg·g^−1^)	19.6 ± 2.5	13.4 ± 2.4	0.040
Nitrate (µg·g^−1^)	23.9 ± 1.9	21.5 ± 2.0	0.429
Ammonium (µg·g^−1^)	45.0 ± 1.7	48.9 ± 5.2	0.226
Available nitrogen (%)	1.2 ± 0.3	1.1 ± 0.1	0.960
Carbon/nitrogen	17.7 ± 4.5	14.9 ± 1.7	0.711

^1^ Mann-Whitney *U* test.

### 3.2. Elaeocarpus joga

The soils away from mature *E. joga* trees contained greater concentrations of Ca, K, Mg, nitrate, and Zn than the soils beneath the trees (Table 2), according to the Mann-Whitney *U* test. The soils beneath *E. joga* trees contained greater concentrations of ammonium, C, Cu, Fe, and N than soils away from these trees (Table 2, Figure 1). Mn, P, and pH were not influenced by sampling location (Table 2). The derived variables percent available nitrogen and C/N were also not influenced by sampling location (Table 2). Net nitrification was greater beneath *E. joga* trees and net mineralization was not influenced by sampling location (Figure 1).

**Table 2.** Proximity to *Elaeocarpus joga* trees and soil chemical traits in Guam. Home soils were collected underneath mature trees. Away soils were collected 30 m and 60 m from focal trees. Means ± SE, n = 8.

Soil trait	Home	Away	Significance
pH	7.6 ± 0.1	7.5 ± 0.1	0.156^1^
Total carbon (mg·g^−1^)	148.3 ± 5.9	92.2 ± 13.3	<0.001
Phosphorus (µg·g^−1^)	151.5 ± 8.8	107.0 ± 15.3	0.135
Potassium (µg·g^−1^)	84.3 ± 11.9	302.0 ± 97.2	<0.001
Calcium (mg·g^−1^)	10.2 ± 0.7	17.8 ± 4.5	0.004
Magnesium (µg·g^−1^)	401.0 ± 21.0	895.0 ± 37.4	<0.001
Manganese (µg·g^−1^)	123.0 ± 6.1	113.0 ± 15.3	0.711
Iron (µg·g^−1^)	51.5 ± 5.7	25.9 ± 4.8	0.002
Copper (µg·g^−1^)	3.2 ± 0.1	1.4 ± 0.2	<0.001
Zinc (µg·g^−1^)	8.3 ± 1.1	13.1 ± 2.5	0.040
Nitrate (µg·g^−1^)	10.3 ± 0.8	24.4 ± 1.1	<0.001
Ammonium (µg·g^−1^)	105.2 ± 3.8	50.8 ± 3.4	<0.001
Available nitrogen (%)	1.0 ± 0.1	1.0 ± 0.1	0.429
Carbon/nitrogen	13.1 ± 0.9	12.4 ± 1.6	0.960

^1^ Mann-Whitney *U* test.

### 3.3. Serianthes nelsonii

The soils away from mature *S. nelsonii* trees contained greater concentrations of P than the soils beneath the trees (Table 3), according to the Mann-Whitney *U* test. The soils beneath *S. nelsonii* trees contained greater concentrations of ammonium, C, Ca, K, Mg, N, and Zn than soils away from these trees (Table 3, Figure 1). Cu, Fe, Mn, nitrate, and pH were not influenced by sampling location (Table 3). The derived variables percent available N and C/N were greater in away soils than in *S. nelsonii* soils (Table 3). The behavioral variables net nitrification and net mineralization were greater beneath *S. nelsonii* trees than away from the trees (Figure 1).

**Table 3.** Proximity to *Serianthes nelsonii* trees and soil chemical traits in Guam. Home soils were collected underneath the only known mature tree remaining on Guam. Away soils were collected 30 m and 60 m from the tree. Means ± SE, n = 8.

Soil trait	Home	Away	Significance
pH	7.3 ± 0.1	7.5 ± 0.1	0.103^1^
Total carbon (mg·g^−1^)	234.3 ± 22.8	99.9 ± 12.2	<0.001
Phosphorus (µg·g^−1^)	74.3 ± 7.8	101.8 ± 16.5	0.040
Potassium (µg·g^−1^)	486.3 ± 32.4	144.8 ± 14.4	<0.001
Calcium (mg·g^−1^)	19.8 ± 1.4	14.4 ± 2.1	0.014
Magnesium (µg·g^−1^)	2763.0 ± 69.4	835.4 ± 61.7	<0.001
Manganese (µg·g^−1^)	130.3 ± 8.4	104.7 ± 19.6	0.226
Iron (µg·g^−1^)	12.3 ± 1.3	20.1 ± 6.7	0.429
Copper (µg·g^−1^)	2.3 ± 0.3	1.7 ± 0.1	0.156
Zinc (µg·g^−1^)	44.8 ± 6.0	15.2 ± 1.9	<0.001
Nitrate (µg·g^−1^)	20.5 ± 1.5	23.1 ± 2.3	0.103
Ammonium (µg·g^−1^)	60.5 ± 3.2	49.1 ± 5.6	0.040
Available nitrogen (%)	0.3 ± 0.1	1.2 ± 0.2	<0.001
Carbon/nitrogen	7.5 ± 0.5	16.4 ± 1.7	<0.001

^1^ Mann-Whitney *U* test.

## 4. Discussion

A contrast in how soils varied beneath *A. mariannensis, E. joga*, and *S. nelsonii* trees in Guam’s limestone forests has been described. As predicted, the *S. nelsonii* trees generated the most profound alterations of habitat biogeochemistry. For example, the concentrations of six of the 10 essential nutrients that were quantified were greater in home soils, and *S. nelsonii* was the only species with soils that differed for both of the calculated variables of percent available N and C/N. The soils beneath *E. joga* trees were different from surrounding soils for eight of the 10 essential elements, but the number of nutrients that were greater in home soils was the same as that in away soils. *Artocarpus mariannensis* trees were associated with soils where all of the important C and N traits were not influenced by sampling location, five of the 10 essential elements were greater in away soils, and only Cu and Zn were greater in home soils. The combined results indicate the magnitude of increased spatial heterogeneity in soil nutrient status for these trees was *A. mariannensis* < *E. joga* < *S. nelsonii.*

Drivers of the spatial changes caused by trees include differences in quality of microsite leaf litter inputs, nutrient inputs from enhanced or reduced animal visitations associated with spatial microsites, nutrients concentrated in stemflow then deposited at the base of individual trees, species-specific vertical and horizontal root contributions, and differential interactions among the tree community with soil microbes. [7,8,26] Leaf litter quality is a function of the ability to absorb and sequester nutrients into leaves throughout their functional life, but also a function of how much of the resident nutrients are re-mobilized and returned to the stems prior to leaf abscission. [27,28] Heterogeneous soil C:N:P relations may profoundly influence the soil microorganism community. [29]

The depauperate soils beneath *A. mariannensis* indicate a high proportion of leaf nutrients may be re-mobilized prior to leaf senescence in these species, and the tree may benefit edaphic consumer guilds with slow-growing conservation growth strategies. The nutrient-rich soils beneath *S. nelsonii* indicate greater leaf nutrient concentrations and lower nutrient re-mobilization during leaf senescence, and the tree may benefit edaphic consumer guilds with fast-growing exploitative growth strategies.

The difference in home and away soils occurred for all three tree species for Ca, K, Mg, and Zn. In contrast, this difference occurred for only one of the three tree species for Fe and Mn. The spatial stability of pH among sites is a function of the buffering capacity for this soil series. The lower horizons are almost pure calcium carbonate. [18]

Studying the abiotic traits of soils that are under the influence of Guam’s sessile, long-lived tree species may improve our ability to conserve the forests and more fully understand the ecosystem services these trees provide. The increased spatial heterogeneity of biogeochemical traits that is delivered by these trees may have profound and lasting influences on soil food web biodiversity in these typhoon forests. This subject deserves further study. More importantly, the increased knowledge provides greater insight into the detrimental effects that are occurring with ongoing losses of these late successional trees due to anthropogenic disturbances.
